# Priorities in Chronic nonbacterial osteomyelitis (CNO) – results from an international survey and roundtable discussions

**DOI:** 10.1186/s12969-023-00851-6

**Published:** 2023-06-30

**Authors:** M. Mohanna, E. Roberts, L. Whitty, J. F. Gritzfeld, C. E. Pain, H. J. Girschick, J. Preston, M. Hadjittofi, C. Anderson, P. J. Ferguson, A. Theos, C. M. Hedrich

**Affiliations:** 1 Department of Women’s and Children’s Health, Institute of Life Course and Medical Sciences, University of Liverpool, Liverpool, UK; 2 Paediatric Rheumatology, Alder Hey Children’s NHS Foundation Trust, Liverpool, UK; 3 Klinik Für Kinder- Und Jugendmedizin, Vivantes Netzwerk Für Gesundheit GmbH, Klinikum Im Friedrichshain, Berlin, Germany; 4 Clinical Health Psychology, Alder Hey Children’s NHS Foundation Trust, Liverpool, UK; 5 Royal Hospital for Children and Young People, Edinburgh, UK; 6 Department of Pediatrics, University of Iowa Stead Family Children’s Hospital, Iowa City, IA USA; 7 Department of Human Science, CRMO Patient/Parent Partner, Georgetown University, Washington, DC USA

**Keywords:** Chronic nonbacterial osteomyelitis, CNO, CRMO, Treatment, Monitoring, Pathophysiology, Diagnosis

## Abstract

**Objective:**

Chronic nonbacterial osteomyelitis (CNO) is an autoinflammatory bone disorder that predominantly affects children and young people. The pathophysiology and molecular mechanisms of CNO remain poorly understood, and diagnostic criteria and biomarkers are lacking. As a result, treatment is empiric and follows personal experience, case series and expert consensus plans.

**Methods:**

A survey was designed to gain insight on clinician and patient experiences of diagnosing and treating CNO and to collate opinions on research priorities. A version containing 24 questions was circulated among international expert clinicians and clinical academics (27 contacted, 21 responses). An equivalent questionnaire containing 20 questions was shared to explore the experience and priorities of CNO patients and family members (93 responses).

**Results:**

Responses were used to select topics for four moderated roundtable discussions at the “International Conference on CNO and autoinflammatory bone disease” (Liverpool, United Kingdom, May 25-26^th^, 2022). The group identified deciphering the pathophysiology of CNO to be the highest priority, followed by clinical trials, necessary outcome measures and classification criteria. Surprisingly, mental wellbeing scored behind these items.

**Conclusions:**

Agreement exists among clinicians, academics, patients and families that deciphering the pathophysiology of CNO is of highest priority to inform clinical trials that will allow for the approval of medications for the treatment of CNO by regulatory agencies.

**Supplementary Information:**

The online version contains supplementary material available at 10.1186/s12969-023-00851-6.

## Introduction

Chronic nonbacterial osteomyelitis (CNO) is an autoinflammatory bone disease that predominantly affects children and young people (CYP) [1]. CNO is characterised by sterile bone inflammation that may result in local swelling, pain, and reduced function. Clinical manifestations cover a spectrum, ranging from mild and sometimes self-limiting mono-focal disease to chronically active or recurrent disease affecting multiple bones. The latter is also referred to as chronic remitting/recurrent multifocal osteomyelitis (CRMO) [2]. The prevalence of CNO was initially estimated to range between 1–2/1,000,000, although newer studies suggested it is higher [3]. A recent German incidence surveillance study estimated annual incidences of 4/1,000,000 [4], and a single-centre study reported disease prevalence to be much higher and comparable to infectious osteomyelitis [5]. While differences between studies may be partially due to geographic and socioeconomic factors, differences in awareness may play a more significant role [6].

Currently, CNO remains a diagnosis of exclusion, with differential diagnoses (among others) including malignancy, infection, metabolic bone disease, and trauma [7]. The absence of diagnostic criteria or disease-specific biomarkers for CNO causes diagnostic delay and missed patients [8]. The observation that signs and symptoms of CNO can be insidious, intermittent and variable make the diagnosis challenging [9].

Imbalanced production of pro- and anti-inflammatory cytokines from innate immune cells is a hallmark in CNO, but the exact molecular underpinnings remain incompletely understood [1]. As a result, treatment of CNO is empiric, targets altered cytokine expression and bone remodelling, and largely relies on expert opinion and retrospective case collections [7]. First-line treatment includes non-steroidal anti-inflammatory drugs (NSAIDs), usually naproxen. In patients with primary involvement of vertebral bodies, especially in the presence of structural damage, second-line treatments are used, including corticosteroids, bisphosphonates, and/or Tumour Necrosis Factor-alpha (TNFα) inhibitors (TNFi) [9]. Anti-infective treatment, though used in most patients before the diagnosis of CNO is established, is ineffective [10].

Over recent years lived experience has been recognised as an invaluable resource and incorporated into research. As a result, patient and public involvement (PPI) has been increasingly recognised as a marker of good research practice [11]. Principles of PPI include the design and focus of research relevant to those affected, involvement of patients at all stages, and ensuring that research is planned and delivered ‘with and by’ rather than ‘on and for’ patients and the public [11, 12]. To share current knowledge, agree on research priorities, and strengthen collaboration, a 2-day meeting was organised in Liverpool, UK, addressing equally CNO patients and their families, charities, clinicians, and academic researchers.

To inform the “International Conference on CNO and Autoinflammatory Bone Disease” (Liverpool, United Kingdom, May 25-26^th^, 2022) and allow actionable outcomes, two online surveys were conducted to explore and compare all stakeholder groups' concerns and research priorities. The survey results facilitated round-table discussions at the face-to-face conference. This manuscript summarises results from the online surveys shared with stakeholder groups and outcomes from round table discussions involving CNO patients and families, clinicians and researchers involved in the field of CNO.

## Materials and methods

### Survey

Two questionnaires were designed to collate experience with diagnosis and treatment, as well as opinions on research priorities among CNO patients and families, health care professionals and CNO researchers. Questionnaires were developed by the Experimental Arthritis Treatment Centre for Children (EACT4Children), involving paediatric rheumatologists from the UK (CMH, CP) and the USA (PJF), the EATC4Children’s manager (JFG) and administrator (LW), a former CNO patient and paediatric trainee (ER), the EATC4Children’s Public Involvement and Engagement (PPIE) manager (JP), and the scientific lead of the CRMO Foundation USA (AT) (Supplements 1 & 2).

A 24-item questionnaire (Supplement 1), designed in 2020, was circulated in 2021 among 27 expert clinicians and clinical academics using *Google Forms*. Proportional representation of North America, the UK, and continental Europe was targeted during the selection of experts which included members of the Childhood Arthritis and Rheumatology Research Association (CARRA), the Paediatric Rheumatology European Society (PReS), the British Society of Rheumatology (Paediatric and Adolescent members) (BSR), and the German Society of Paediatric and Adolescent Rheumatology (GKJR). Proportional representation of North America, the UK, and continental Europe were targeted during the selection of experts. All experts were consultant-grade clinicians who diagnose and treat a minimum of 10 patients with CNO per year and have clinical experience with CNO for > 5 years. All have published work on CNO's clinical and/or pathophysiological aspects with an impact factor > 4 within the past five years (2016–2021). Experts who responded proportionally represented geographic regions as follows: seven from North America, seven from the UK, and seven from continental Europe.

A second 20-item questionnaire was designed using lay language to explore the experience and priorities of CNO patients and family members (Supplement 2). The survey was circulated internationally (*Google Forms*) in 2021 via the EATC4Children’s social media pages (Facebook: 50 followers, Twitter: 618 followers), the UK CRMO Facebook group (486 members), the CRMO Foundation USA (Facebook: 1300 followers, Twitter: 284 followers), and individuals at European Network for Children with Arthritis (ENCA) and Rare Autoinflammatory Conditions Community (RAAC-UK) who distributed it to their patient contacts/groups.

Both questionnaires included qualitative and quantitative questions. For analysis, quantitative data were grouped, and graphics were produced to summarise differences and similarities between clinicians’ versus patients’ experiences and priorities. A thematic analysis of free-text data was conducted by two investigators (MM and ER) to collate the information and highlight key areas of concern and research priorities. The efforts of the two investigators were then compared and moderated (CMH and PJF) to confirm themes.

### Roundtable discussion

Results from the questionnaires, as outlined in this report, were used to assign topics for four moderated roundtable discussions at the “International Conference on CNO and autoinflammatory bone disease” in Liverpool, May 25^th^-26^th^, 2022. Facilitated discussion topics included: i) Clinical Trials (facilitators: CP, ER), ii) Pathophysiology and Molecular Mechanisms (PF, AT), iii) Mental and Emotional Well-being (MH, MM), and iv) (to allow tracing and comparison of future studies) Nomenclature (HJG, CA). Roundtable discussion participants included patients and families (N ~ 15), academics, and clinical staff (consultants, medical subspecialty trainees, specialist nurses) (N ~ 30). Moderation by facilitators was minimal to allow for free and uninterrupted discussion amongst parties. It included agreeing a framework for discussion (e.g. discussing Population, Intervention, Control, and Outcomes in the “clinical trials” discussion), reminding participants of timing of session, note-keeping and encouragement of contribution from all parties. At the end of each discussion session, facilitators moved from table to table, so all participants discussed all topics (20 min each). Results were recorded and presented to the groups after the subsequent conference sessions.

## Results

### Survey

**Fig. 1:**
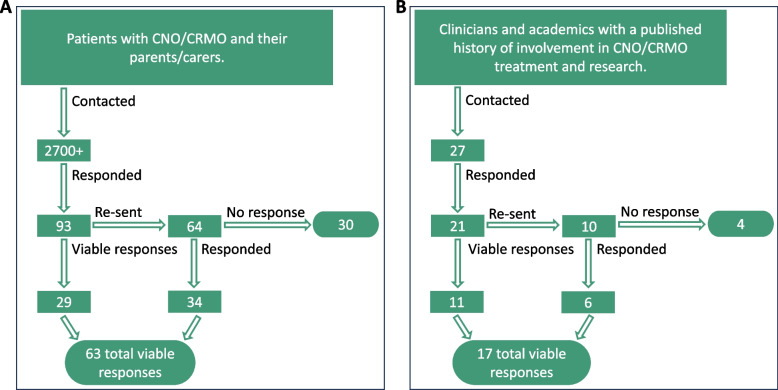
**Survey sharing strategy and responses.** Surveys addressing patients/families (**A**) and clinicians/academics (**B**) were shared through email lists and online groups as indicated. For one question, initially, not all responses were usable. Thus, in addition to 29 initially viable responses from patients and 11 from clinicians/academics, more usable responses were collected through contacting individuals who provided their email contacts. A total of 34 additional viable responses were collected from patients/families (63 total), and 6 additional viable were received from clinicians/academics (17 total)

There were 93 responses to the patient questionnaire (Fig. 1A)*.* Of these, 23% (21/93) were from children and young people (CYP) affected by CNO (Age range: 8–31 years, median age: 20 years), and 77% (72/93) were from parents or carers on behalf of the young person they care for (on behalf of children and young people aged 5–20 years, median: 12 years) (S1Q2, Q3) (Supplement 3A)*.* Of patient/carer responses, 58% (54/93) were from North America, 21% (19/93) were from the UK and Ireland, 11% (10/93) were from continental Europe, and 10% (10/93) were from other regions (including Australia, New Zealand, Central and South America) (S1Q4).

A total of 21 expert clinicians and clinical academics responded to the questionnaire (21/27: 78%, Fig. 1B). Of these, 71% (15/21) had been practising as a specialist rheumatologist for > 10 years (S2Q5). All respondents treated paediatric patients, with 19% (4/21) also treating adults (S2Q6). Responses from clinicians/clinical academics were equally distributed between North America, the UK, and continental Europe (all 7/21, 33.3%) (S2Q3) (Supplement 3B)*.*

**Fig. 2:**
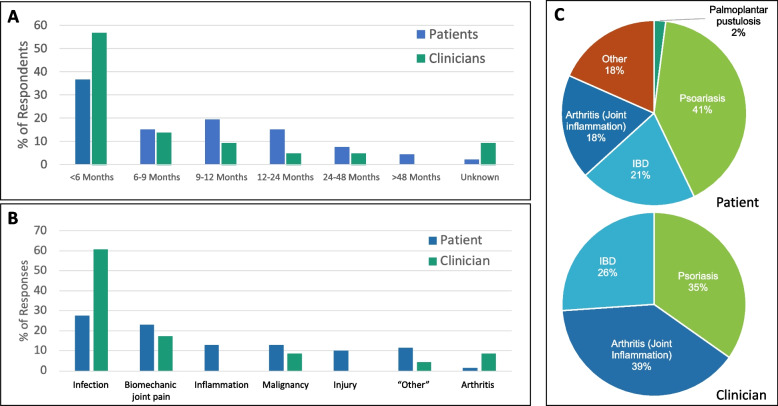
**Experience with diagnosis and comorbidities.** **A** Asked about time to diagnosis, patients and carers reported a median time to diagnosis of approximately 6 months (mean: 14 months). Clinicians/clinical academics estimated a median and mean of 6 months. **B** 56% of CYP/carers reported they had received an alternative diagnosis prior to correct diagnosis. Most common initial diagnoses included osteoarticular infections, biomechanical joint pain, “inflammation”, and cancer. Among expert clinicians/clinical academics, 100% reported that, in their experience, CNO patients receive an incorrect working diagnosis before CNO is diagnosed, with the most common being infectious causes*.* **C** Of all CYP/carers responding, 46% reported inflammatory conditions alongside CNO; 41% of these patients experienced psoriasis, 21% had inflammatory bowel disease (IBD), and 18% had arthritis. All clinicians had treated patients with an additional inflammatory condition, the most common being arthritis, followed by psoriasis and IBD

The patient/carer questionnaire asked about their experience with time to (correct) diagnosis, initial incorrect diagnoses, and clinical phenotypes and treatment, and the clinicians/clinical academics were asked about their experience in practice with the aforementioned aspects of CNO (Fig. 2). Patients and carers reported a median time to diagnosis of approximately 6 months, with a mean of 14 months (S1Q8). Clinicians/clinical academics estimated a median and mean of 6 months (S2Q11) (Fig. 2A). As many as 56% of CYP/carers reported they had received an alternative diagnosis before they were diagnosed with CNO (S1Q9). The most common initial diagnoses included osteoarticular infections (28%), biomechanical joint pain (23%), “inflammation” (13%), and cancer (13%) (S1Q10) (Fig. 2B). Among expert clinicians/clinical academics, 100% reported that, in their experience, CNO patients receive an incorrect working diagnosis before CNO was diagnosed (S2Q13), with the most common being infectious causes (61%) (S2Q13) (Fig. 2B)*.*

Because CNO can be associated with inflammatory involvement of extraosseous structures that can aid in diagnosing CNO, CYP/families and clinicians were asked about their experience with these. Of all CYP/carers responding, 46% reported inflammatory conditions alongside CNO (S1Q12); 41% of these patients experienced psoriasis, 21% had inflammatory bowel disease (IBD), and 18% had arthritis (S1Q13) (Fig. 2C). All clinicians had treated patients with an additional inflammatory condition (S2Q14), the most common being arthritis (39%), followed by psoriasis (35%) and IBD (26%) (S2Q14) (Fig. 2C).

**Fig. 3:**
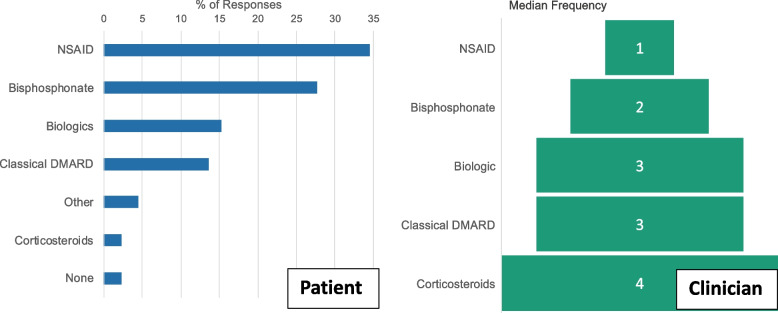
**Experience with treatment.** Among CYP, the most common medications taken were NSAIDs (34%), followed by bisphosphonates (28%), biological (15%), and conventional (14%) disease-modifying antirheumatic drugs (DMARDs). Clinicians shared views of what patients had reported

Treatment of CNO is empiric and not standardised across centres, and therefore is largely based on the personal experience of the treating clinician, case series, and expert opinion [8]. Therefore, Patients/carers and clinicians were surveyed about their experience with treatment choices. Among CYP/carers responding to the survey, the most common medications taken were nonsteroidal anti-inflammatory drugs (NSAIDs) (34%), followed by bisphosphonates (28%), biological (15%), and conventional (14%) disease-modifying antirheumatic drugs (DMARDs) (S1Q14). Clinicians were asked to rank medications, with one being the medication they most frequently prescribed for their patients with CNO. Responses were in close agreement with patient/carer experience (Fig. 3) (S2Q16).

**Fig. 4:**
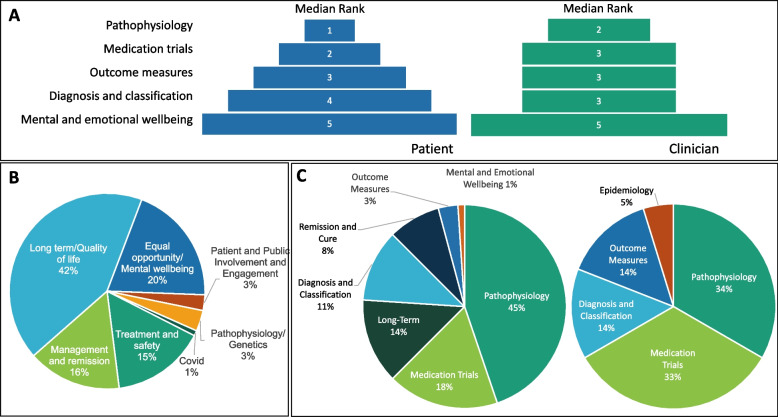
**Identification of research priorities.** **A** Patients/carers and clinicians were asked to rank research topics/areas of interest (namely: pathophysiology, medication trials, outcome measures, diagnosis and classification criteria, mental and emotional well-being) to allow future prioritisation of joint efforts. **B** Furthermore, open questions were asked, allowing free text responses. **C** Final themes for roundtable discussions were identified from free-text responses. Notably, research priorities aligned closely with the five topics previously ranked, including ‘Investigations into the underlying causes of CNO (Pathophysiology)’, ‘Medication trials testing drugs’, ‘Defining the ways we diagnose and classify CNO’, ‘Finding outcome measures that help us to treat patients more effectively in clinic, and research studies’, and ‘Studies into how the disease affects your mental and emotional wellbeing’

Lastly, patients/carers and clinicians were asked to rank research topics/areas of interest (namely: pathophysiology, medication trials, outcome measures, diagnosis and classification criteria, mental and emotional well-being) to allow future prioritisation of joint efforts. While both groups agreed on the highest priority of deciphering the pathophysiology of CNO, clinicians did not come to an agreement on how to rank closely related medication trials, outcome measures, and diagnosis and classification criteria (Fig. 4A). Both groups ranked impacts on emotional and mental well-being as their lowest research priority (S1Q15, S2Q17). Notably, this question had to be re-shared with a subset of respondents (both patients/carers and clinicians/academics) with additional clarification. The question was worded in a way that a subset of respondents responded in a different manner than intended. Several respondents isolated each topic and rated them each out of 5, resulting in incomparable data. Thus, we redistributed this question to those who did not answer as intended, hence giving a lower number of respondents to this specific item (Fig. 1).

In addition to the ranking exercise, open questions were asked, allowing free text responses. Patients/carers were asked what their biggest concerns were regarding the impact of CNO on their well-being. Most commonly occurring themes were ‘long-term outcomes and future quality of life when living with CNO’ (42%), followed by both ‘treatment choice and safety including side effects’ (16%) and ‘disease management monitoring of response and remission’ (16%) (Fig. 4B) (S1Q17).

From aforementioned responses to questionnaires, final themes for roundtable discussions, including free-text responses. Notably, research priorities aligned closely with the five topics previously ranked, including ‘Investigations into the underlying causes of CNO (Pathophysiology)’ (patients/carers: 45%, clinicians: 34%), ‘Medication trials testing drugs’ (patients/carers: 18%, clinicians: 33%), ‘Defining the ways we diagnose and classify CNO’ (patients/carers: 11%, clinicians: 14%), ‘Finding outcome measures that help us to treat patients more effectively in clinic, and research studies’ (patients/carers: 3%, clinicians: 14%), and ‘Studies into how the disease affects your mental and emotional wellbeing’ (patients/carers: 1%, clinicians: N/A), (Fig. 4C*)*. The top 3 themes were chosen for roundtable discussions.

Considering variable nomenclature for CNO across the existing literature, an additional topic ‘nomenclature’ was added by the organizers.

### Roundtable discussions

**Fig. 5:**
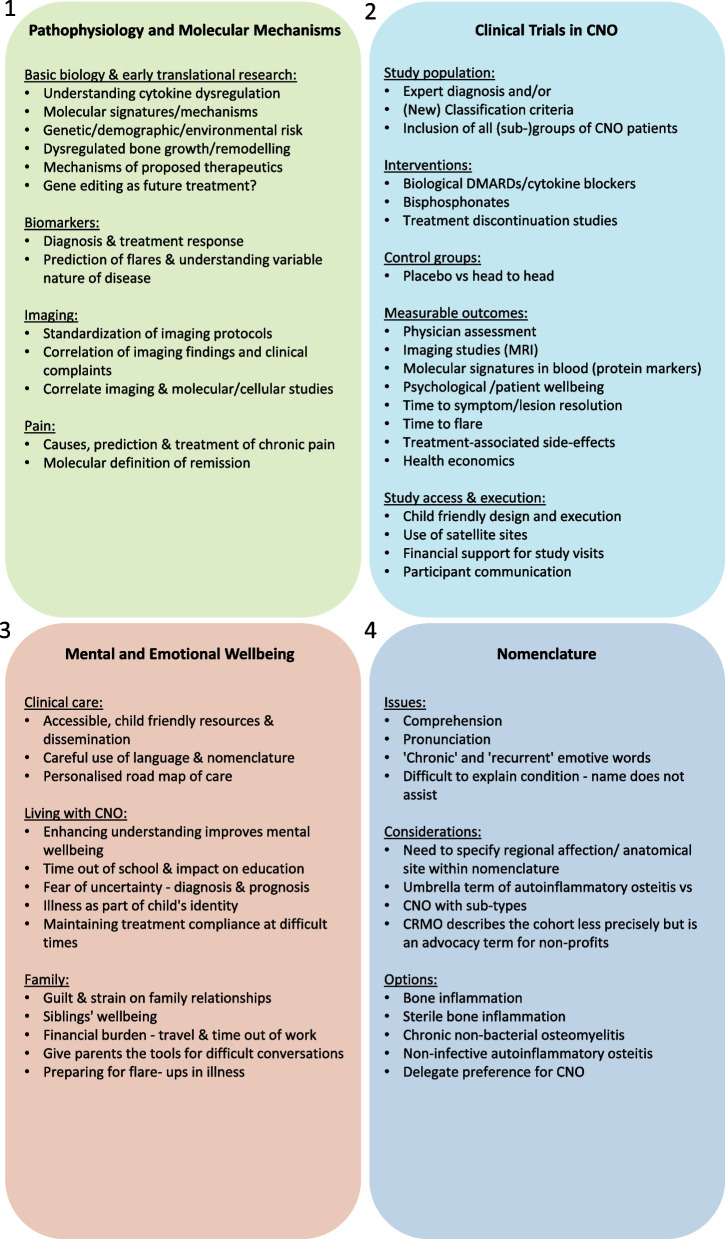
**Outcomes from roundtable discussions.** Aforementioned topics were discussed at a face-to-face meeting in Liverpool, involving expert clinicians, clinical academics, patients and carers. Outcomes of moderated discussions are displayed

The four aforementioned topics were discussed at a face-to-face meeting in Liverpool, involving expert clinicians, clinical academics, patients and carers. Discussions were moderated and recorded, and outcomes are outlined below (Fig. 5).

1. Pathophysiology and molecular mechanisms

The discussion around pathophysiology and molecular mechanisms of CNO was extensive and detailed, with themes extending from individual predisposition/susceptibility to associated long-term outcomes. Equally, patients, carers and experts recognised the vast need for research into this field. Parents/carers wish for a greater understanding of modifiable factors that may allow for disease prevention or early and “mild” interventions, i.e., dietary interventions, smoking, geographic variability, etc. The urgent need for collaboration was recognized, especially considering logistical issues around sharing comparable samples, i.e., sample handling, shipment, variability in reagents used.

2. Clinical trials in CNO

Discussions around designing clinical trials for patients with CNO naturally considered the ‘PICO’ framework, discussing Population, Intervention, Controls and Outcome measures. Most participants felt that trials should, ideally, be accessible to all CNO patients and not limited to sub-cohorts, e.g., only those with multifocal bone disease. However, it was agreed that some CNO patients may be harder to study - i.e., those with vertebral fractures, where treating with NSAIDs alone as a comparator may be considered unethical. Consideration was given to classification of CNO patients and eligibility criteria, reflecting diagnostic challenges and lack of criteria used for diagnosis in clinical practice. Soon to be published suggestions for EULAR/ACR criteria were agreed as a valuable tool [13]. Discussion around appropriate interventions was broad, likely reflecting the lack of clinical trials in this field. Interestingly there were differing opinions between CYP, parents and clinicians regarding controls. Families favoured the generation of evidence for the efficacy of current and future treatments in placebo-controlled trials, whilst clinicians expressed their preference for head-to-head comparisons. This was based on their concerns around ongoing pain and damage accrual in the placebo group. There was a unanimous understanding that the measured outcomes, including physical exam, biomarkers and imaging, were required to be patient centred. It was recognised that outcome measures important to patients and families (particularly pain, sleep, health economics, school attendance) are difficult to quantify and validate reliably. Thus, additional objective tools must be included. Acknowledging the current lack of prospectively and independently validated scoring tools is important for future research in the field of CNO and in paediatrics in general.

3. Mental and emotional wellbeing

This round table was an opportunity for all delegates to share their experiences of living with or caring for people with CNO. Many common issues were shared. Families emphasized uncertainties of diagnosis, many referring to frightening conversations where the differential diagnosis of malignancy was discussed. The insidious and relapsing/remitting pattern of the disease was also acknowledged, with many patients/carers expressing frustrations around the unpredictable course of symptoms and having to explain to others why their levels of functional ability may vary depending on whether they are experiencing flares or remission. Other themes included helping children/patients to understand their diagnosis, and the implications for the wider family, including emotional and financial impacts.

4. Nomenclature

Varying nomenclature was a concern equally for clinicians/scientists and patients and their families. Clinicians recognised the importance of nomenclature for classification, both for diagnostic purposes and for future laboratory and clinical research. For example, the acronym CRMO may disadvantage patients with monofocal disease in their access to certain/future treatments. Parents/patients felt the use of the word ‘chronic’ sounded “scary”, and took away hope for recovery, commenting that many other chronic diseases do not reference chronicity in name, e.g., asthma or epilepsy. Overall, there was consensus that CNO may be a good "umbrella term” and that sub-type nomenclature may be of use, especially because some sub-groups of CNO require more “aggressive” management plans, e.g., CNO of the jaw. Because of its inclusivity, there was an overall preference for CNO. Because families and some charities use CRMO as a “brand”, the combination of “CNO/CRMO” may be useful when publishing data.

## Discussion

This initiative brought together opinions and experiences of patients, carers/families, clinicians, academics, and charity representatives from across the world and culminated in setting a list of priorities to focus research into CNO.

While, overall, experience and opinions largely overlapped between patients/carers and clinicians, one interesting and unexpected difference was observed. All clinicians reported to exclusively care for patients who had received an incorrect diagnosis before CNO, whilst only 56% of patients reported experiencing misdiagnosis. This difference could be due to working diagnoses not being considered ‘misdiagnoses’ by patients, or a lack of communication with patients regarding diagnostic uncertainty during the investigation*.* The most common misdiagnosis mentioned by both groups was infection, including infective osteomyelitis and arthritis. In agreement with the published literature, a broad range of additional differential diagnoses was reported, including biomechanical joint pain, injury, inflammation, and malignancy [7, 14–16].

Diagnosis may be informed by the presence of CNO-associated extra-osseous inflammation which was present in almost half of all patients responding to the survey. Indeed, soon to be published EULAR/ACR classification criteria for CNO also include associated organ involvement [13]. Notably, it is likely that overlapping features between CNO and associated diseases blurs lines when it comes to diagnosis and treatment and may have affected aforementioned discrepancies in the response to diagnostic delay and false diagnoses [2, 17, 18].

Research priorities showed alignment between patients and clinicians in both surveys and round table discussions. However, ranking of priorities by clinicians was slightly less “clear” when compared to patients/carers, and delivered “pathophysiology” as highest priority as it may allow individualized and target-directed treatments, followed by “medication trials”, “outcome measures” and “diagnosis and classification” as shared next priorities. The high priority of studies investigating the molecular pathophysiology of CNO may be explained by its potential to inform several aspects affecting patients and carers, including the development of biomarkers that may allow for early diagnosis and treatment initiation, and the development of effective, target-directed and individualized treatments [2]. Clinicians/academics may, furthermore, not have been able to distinctly rank “medication trials”, “outcome measures” and “diagnosis and classification” because of their related character and the fact that the definition of representative study populations and outcome measures are necessary for the delivery of clinical trials and laboratory studies. In line with outcomes from this initiative, an expert consensus meeting following the “International Conference on CNO and Autoinflammatory Bone Disease” suggested use of expert diagnosis and meeting EULAR/ACR classification criteria for CNO [13] as inclusion criteria for clinical trials [19].

It appears surprising that only relatively few responders to the survey identified mental and emotional impacts on patient wellbeing as the highest priority. This may have been caused by an understanding amongst all stakeholders that an improved understanding of disease mechanisms and better treatments will reduce negative impacts on mental health. In addition, the mental and emotional impact may have been seen as a therapeutic priority, rather than a research priority. Treatment trials, outcome measures, and diagnosis and classification criteria are each closely related areas of research and rely on each other, likely factoring into the difficulty in ranking their order of priority.

Despite mental and emotional well-being initially ranking lowest in the order of research priorities, there were many critical points raised at the round table, particularly from patients and loved ones of those suffering. One was that the stigma surrounding the invisible nature of the illness is perpetuated by having good days and can lead to the disease becoming a part of the child’s identity and a sense that they must prove themselves. Interestingly, the literature reports differences in perceived health-related quality of life and objective measures of physical activity amongst CNO patients, further suggesting that support for mental wellbeing is important for these patients [20]. Dealing with such things at a young age also created strain within the family unit, and the emotional well-being of those around them, most importantly siblings, who may be affected just as much but perhaps in different ways.

Collating patient/carer opinion alongside clinician opinion and facilitating mixed groups for the roundtable discussions is a particular strength of this priority setting exercise. Informed by feedback to surveys, during the face-to-face meeting, round-table discussions started broad and were delivered with enthusiasm, narrowing down to actionable research priorities that will benefit patients with CNO and their families. Two members of the organisation team, MM (medical student) and ER (junior doctor), were relatively close in age to the patient population. Their involvement, particularly in facilitating the round-table discussions at the conference, was a key enabler of the young persons’ participation. Involvement in this research as a medical student has enhanced both the research findings and student learning, as has been previously noted [12].

Limitations of this exercise are caused largely by survey bias with the sample of patient/carer responses being small relative to the estimated prevalence of CNO (1–2 million patients) [3]. Notably, a majority of those contacted already were involved with CNO support groups and/or research. Therefore, patients and carers less connected to the community had less opportunity to contribute to the discussion. The survey was most accessible to individuals able to read English with access to sites of publications (e.g., social media groups). Whilst these respondents may not differ in any significant way from the population overall, limitations could in the future be resolved by widening outreach to participants to avoid potential for the creation of “echo chambers”.

Lastly, questionnaires provide easily curatable and comparable quantitative data and are more likely to get a greater number of responses [21, 22]. Especially in times of the COVID-19 pandemic, electronic questionnaires were easier and safer to distribute. The impersonal format, however, may have made it difficult for an individual person’s comment to spark ideas and corroboration amongst the group [23]. Offering the choice to participate via questionnaire or interview may have increased participation rates and avoided confusion but could have created challenges in comparing data via these two modes of collection [24]. Although the questionnaire was shared internationally, the meeting with face-to-face round-table discussions took place in Liverpool, UK. Whilst much of the meeting was broadcast via an online meeting platform (Zoom), the round-table discussions were held in person only. While financial support for travel and accommodation was offered where possible, this limited accessibility.

## Conclusions

Based on an international survey comprised of two questionnaires, and moderated round-table discussions including patients, carers, clinicians and academics, research priorities in CNO have been identified. Collaborative efforts are necessary to deliver meaningful research to understand the pathomechanisms of CNO and develop disease biomarkers and target-directed treatments. Clinical trials are urgently needed to generate evidence for safe and effective treatment and receive regulatory approval. Mental wellbeing is heavily impacted by delayed diagnosis, uncertainty around disease course, and lack of individualized treatment.

## Supplementary Information

**Additional file 1**.

**Additional file 2.**

**Additional file 3.**

## Data Availability

Information shared in the manuscript can be accessed based on reasonable request.

## Abbreviations

ACR: American College of Rheumatology
BSR: British Society of Rheumatology
CARRA: Childhood Arthritis and Rheumatology Research Association
CNO: Chronic nonbacterial osteomyelitis
CRMO: Chronic recurrent multifocal osteomyelitis
CYP: Children and young people
DMARD: Disease-modifying antirheumatic drugs
EACT4Children: Experimental Arthritis Treatment Centre for Children
ENCA: European Network for Children with Arthritis
EULAR: European League Against Rheumatism
GKJR: Society of Paediatric and Adolescent Rheumatology, German speaking
N/A: Not applicable
NSAID: Nonsteroidal anti-inflammatory drug
PICO: Population, Intervention, Controls and Outcome
PPI: Patient and public involvement
PReS: Paediatric Rheumatology European Society
RAAC-U: Rare Autoinflammatory Conditions Community, United Kingdom
TNFα: Tumour Necrosis Factor-alpha
TNFi: Tumour Necrosis Factor inhibitors
