# Supplementary material for: Priorities in Chronic nonbacterial osteomyelitis (CNO) – results from an international survey and roundtable discussions
Source: Pediatr Rheumatol Online J. 2023 Jun 30;21:65. doi: 10.1186/s12969-023-00851-6 (PMC10311767; doi:10.1186/s12969-023-00851-6)
Supplement: Supplementary file 1 — Additional file 1. [file 12969_2023_851_MOESM1_ESM.pdf]

Supplement 1

5th International Meeting on Chronic Nonbacterial Osteomyelitis and Autoinflammatory Bone Disease

After a two-year delay, we are now happy to be able to host the first ever international Chronic Nonbacterial Osteomyelitis (CNO)/Chronic Recurrent Multifocal Osteomyelitis (CRMO) patient/family-driven research conference on May 25-26th, 2022 at the University of Liverpool, UK.

As part of this, we want to understand the key CNO/CRMO questions that you; the patients, and families see as the top priorities for getting some answers through research studies.

We have put together a few questions to collect this information from CNO/CRMO patients and families. The responses to the questions will then be used to set the agenda for the conference.

Please file one response per family. Children under 12 should complete the questionnaire together with a parent/carer.

You will be able to register your interest in attending the conference on our website www.EATC4Children.co.uk in the new year. Registration fees are waived for patients/parents but places are limited (and this does not include travel or accommodation costs).

Should you have any questions please email: laura.whitty@liverpool.ac.uk. We look forward to working together to better understand this rare disease.

* Required

1. Name

____

2. Are you a young person with CNO/CRMO or a parent/carer looking after a child (between the ages of 5-18) with this condition?

   Mark only one oval.

   ◯ Young person
   ◯ Parent/Carer

3. How old are you/how old is your child?

4. Where do you live? *

Mark only one oval.

- UK/Ireland
- Continental Europe
- North America
- Central/South America
- Africa
- Asia
- Australia/New Zealand
- Prefer not to answer

5. Did you fill this survey in previously (Feb 2020)? *

Mark only one oval.

- Yes
- No

6. Are you happy to have your responses to this survey published in a manuscript summarizing patient and families needs? Your responses would be fully anonymised prior to publication. There would be no identifying information included. *

Mark only one oval.

- Yes
- No

7. How many years has it been since diagnosis?

____

8. How long did it take to receive a diagnosis?

____

9. Were you/your child (wrongly) diagnosed with another condition before the diagnosis CNO/CRMO was made?

Mark only one oval.

- ◯ Yes
- ◯ No

10. If you answered yes, what was the diagnosis?

____
____
____
____
____

11. Where are you/your child treated?

Mark only one oval.

- ◯ Outpatient practice
- ◯ Community hospital or health centre
- ◯ Tertiary care centre (e.g. University Medical Centre)
- ◯ Other: _______________________

12. Do you/your child have any other inflammatory symptoms/conditions?

Mark only one oval.

- ◯ Yes
- ◯ No

13. If you answered yes, which condition?

Mark only one oval.

- ◯ Palmoplantar pustulosis
- ◯ Psoriasis
- ◯ Inflammatory bowel disease (Crohn's disease or ulcerative colitis)
- ◯ Arthritis (joint inflammation)
- ◯ Other: ______________________

14. Please tick what medications you/your child is currently taking for CNO/CRMO? *

Check all that apply.

- ☐ NSAID (e.g. naproxen, ibuprofen, etc.)
- ☐ Corticosteroids (e.g. prednisolone, prednisone, etc.)
- ☐ TNF inhibitor (e.g. etanercept, adalimumab, infliximab, etc.)
- ☐ Bisphosphonate (e.g. pamidronate, zoledronic acid, etc.)
- ☐ Classical DMARD (methotrexate, sulfasalazine, etc.)
- ☐ Other: ______________________

15. What do you think is the biggest research priority for CNO/CRMO? (Please rank them - 1 being the highest priority) *

Mark only one oval per row.

	1	2	3	4	5
**Investigations into the underlying causes of CNO/CRMO (Pathophysiology)**	⬭	⬭	⬭	⬭	⬭
**Medication trials testing drugs**	⬭	⬭	⬭	⬭	⬭
**Studies into how the disease affects your mental and emotional wellbeing**	⬭	⬭	⬭	⬭	⬭
**Finding outcome measures that help us to treat patients more effectively in clinic and research studies**	⬭	⬭	⬭	⬭	⬭
**Defining the ways we diagnose and classify CNO/CRMO**	⬭	⬭	⬭	⬭	⬭

16. Is there a research question(s) you have about CNO/CRMO that you would like an answer to? *

17. What is your biggest concern about your/your child's CNO/CRMO? *

18. If you are UK based, would you be interested in attending a 2-day conference to learn about and discuss CNO/CRMO research? *

Mark only one oval.

- Yes
- No
- N/A

19. If you answered yes, would you be willing to share your/your family's story and experiences with CNO/CRMO at the conference (support would be provided to you)? *

Mark only one oval.

- Yes
- No
- N/A

20. Can we contact you about this? Please enter your email below.

This content is neither created nor endorsed by Google.

Google Forms
