# Supplementary material for: Priorities in Chronic nonbacterial osteomyelitis (CNO) – results from an international survey and roundtable discussions
Source: Pediatr Rheumatol Online J. 2023 Jun 30;21:65. doi: 10.1186/s12969-023-00851-6 (PMC10311767; doi:10.1186/s12969-023-00851-6)
Supplement: Supplementary file 2 — Additional file 2. [file 12969_2023_851_MOESM2_ESM.pdf]

Supplement 2

5th International Meeting on Chronic Nonbacterial Osteomyelitis and Autoinflammatory Bone Disease

After a two-year delay, we are now happy to be able to host the first ever international Chronic Nonbacterial Osteomyelitis (CNO)/Chronic Recurrent Multifocal Osteomyelitis (CRMO) patient/family-driven research conference on May 25-26th, 2022 at the University of Liverpool, UK.

As part of this, we want to understand the key CNO/CRMO questions that you see as the top priorities for getting some answers through research studies.

We have put together a few questions to collect this information from CNO/CRMO clinicians. The responses to the questions will then be used to inform the agenda for the conference.

Please ensure that you complete this survey ONCE only.

Should you have any questions please email: laura.whitty@liverpool.ac.uk. We look forward to working together to better understand this rare disease.

* Required

1. Name *

____

2. Institution *

____

3. Where do you live? *

Mark only one oval.

- UK/Ireland
- Continental Europe
- North America
- Central/South America
- Africa
- Asia
- Australia/New Zealand
- Prefer not to answer

4. What is your sub-speciality? *

5. How long have you been practicing as a specialised rheumatologist? *

Mark only one oval.

- 0-3 years
- 3-5 years
- 5-10 years
- Over 10 years

6. Do you work with adults or children? *

Mark only one oval.

- ( ) Adults
- ( ) Children
- ( ) Both

7. How many CNO/CRMO patients do you diagnose per year? *

____

8. How many CNO/CRMO patients do you currently treat at your institution? *

____

9. Where do you treat your patients? *

Mark only one oval.

- ( ) Outpatient practice
- ( ) Community hospital or health centre
- ( ) Tertiary care centre (e.g. University Medical Centre)
- ( ) Other: _______________

10. Are you happy to have your responses to this survey published in a manuscript * summarizing patient and families needs? Your responses would be fully anonymised prior to publication. There would be no identifying information included.

Mark only one oval.

- ( ) Yes
- ( ) No

11. How long would you estimate the average CNO/CRMO patient waits for a correct diagnosis after symptom onset? *

12. Were any of your patients (wrongly) diagnosed with another condition before the diagnosis CNO/CRMO was made? *

Mark only one oval.

- Yes
- No

13. If you answered yes, what was the most common diagnosis?

14. Do your patients have any other inflammatory symptoms/conditions? *

Mark only one oval.

- Yes
- No

15. If you answered yes, which condition is most common?

Mark only one oval.

- Palmoplantar pustulosis
- Psoriasis
- Inflammatory bowel disease (Crohn's disease or ulcerative colitis)
- Arthritis (joint inflammation)
- Other: ____________

16. Please tick what medications you prescribe most commonly for CNO/CRMO? *
(Please rank by prescription frequency - 1 being most frequent)

Mark only one oval per row.

	1	2	3	4	5
**NSAID (e.g. naproxen, ibuprofen, etc.)**	⬭	⬭	⬭	⬭	⬭
**Corticosteroids (e.g. prednisolone, prednisone, etc.)**	⬭	⬭	⬭	⬭	⬭
**TNF inhibitor (e.g. etanercept, adalimumab, infliximab, etc.)**	⬭	⬭	⬭	⬭	⬭
**Bisphosphonate (e.g. pamidronate, zoledronic acid, etc.)**	⬭	⬭	⬭	⬭	⬭
**Classical DMARD (methotrexate, sulfasalazine, etc.)**	⬭	⬭	⬭	⬭	⬭

17. What do you think is the biggest research priority for CNO/CRMO? (Please rank them - 1 being the highest priority) *

Mark only one oval per row.

	1	2	3	4	5
**Investigations into the underlying causes of CNO/CRMO (Pathophysiology)**	◯	◯	◯	◯	◯
**Medication trials testing drugs**	◯	◯	◯	◯	◯
**Studies into how the disease affects your mental and emotional wellbeing**	◯	◯	◯	◯	◯
**Finding outcome measures that help us to treat patients more effectively in clinic and research studies**	◯	◯	◯	◯	◯
**Defining the ways we diagnose and classify CNO/CRMO**	◯	◯	◯	◯	◯

18. Is there a research question(s) you have about CNO/CRMO that you feel needs to be addressed? *

19. Would you be interested in attending a 2-day conference to discuss CNO/CRMO research? *

Mark only one oval.

- ( ) Yes
- ( ) No

20. Can we contact you with further information about the conference? If so, please enter your email below. *

____

This content is neither created nor endorsed by Google.

Google Forms
