# Supplementary material for: Priorities in Chronic nonbacterial osteomyelitis (CNO) – results from an international survey and roundtable discussions
Source: Pediatr Rheumatol Online J. 2023 Jun 30;21:65. doi: 10.1186/s12969-023-00851-6 (PMC10311767; doi:10.1186/s12969-023-00851-6)
Supplement: Supplementary file 3 — Additional file 3. [file 12969_2023_851_MOESM3_ESM.pdf]

A
B

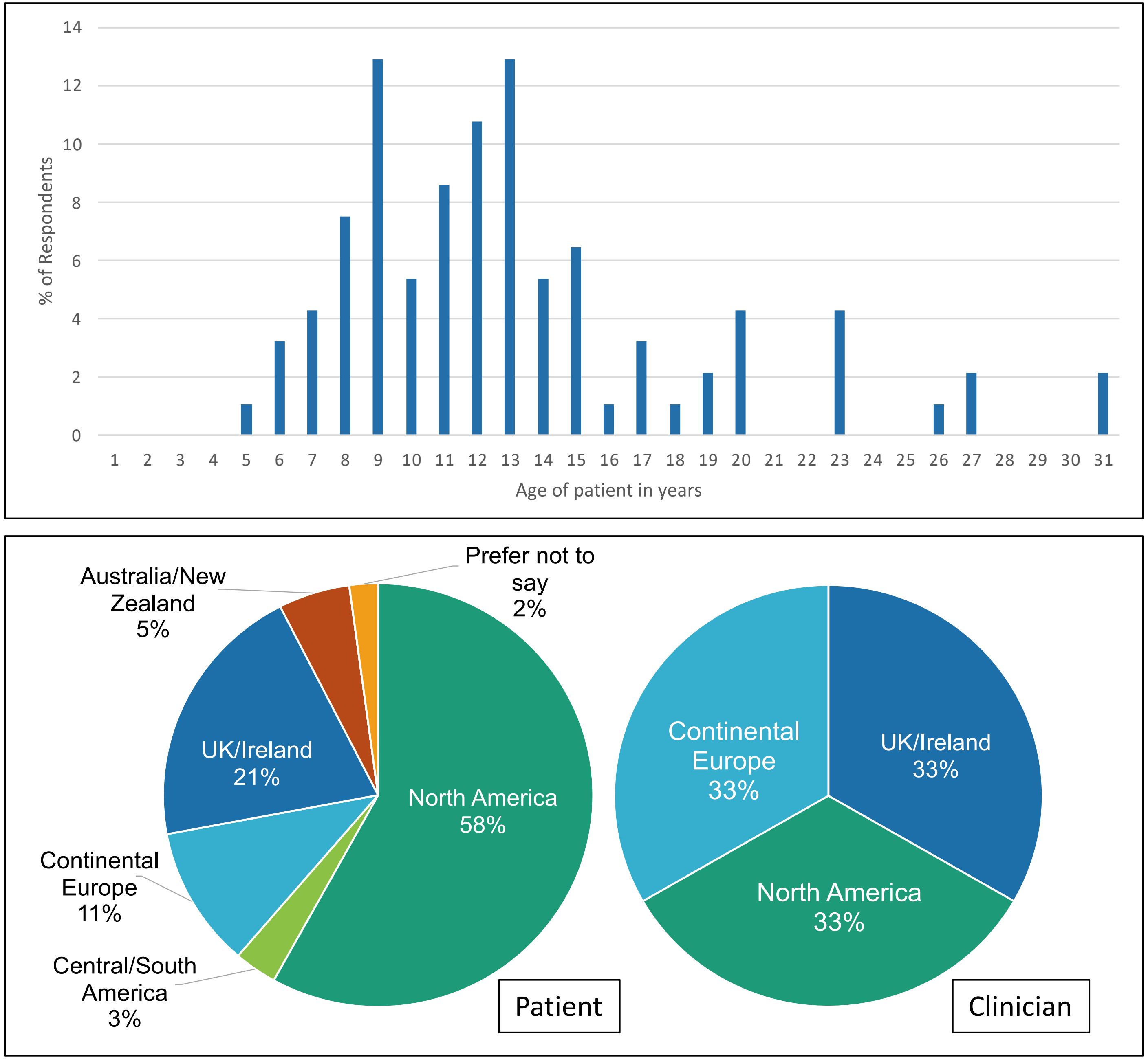

<u>Supplement 3:</u> Survey demographics. A) There were 93 responses to the patient questionnaire, 23% (21/93) were from children and young people (CYP) affected by CNO, and 77% (72/93) were from parents/carers. The ages of the patients ranged from 5-31 years (Median: 12). B) Of patients/carers, 58% (54/93) were from North America, 21% (19/93) were from the UK and Ireland, 11% (10/93) were from continental Europe, and 10% (10/93) were from other regions (including Australia, New Zealand, Central and South America). Responses from clinicians/clinical academics were equally distributed between North America, the UK, and continental Europe (all 7/21, 33.3%).

Supplement 3
